# Increased risk of tinnitus in patients with chronic kidney disease: A nationwide, population-based cohort study

**DOI:** 10.1371/journal.pone.0183192

**Published:** 2017-08-15

**Authors:** Cheng-Ping Shih, Hung-Che Lin, Chi-Hsiang Chung, Po-Jen Hsiao, Chih-Hung Wang, Jih-Chin Lee, Wu-Chien Chien

**Affiliations:** 1 Department of Otolaryngology-Head and Neck Surgery, Tri-Service General Hospital, National Defense Medical Center, Taipei, Taiwan, ROC; 2 School of Public Health, National Defense Medical Center, Taipei, Taiwan, ROC; 3 Department of Medical Research, Tri-Service General Hospital, Taipei, Taiwan, ROC; 4 Division of Nephrology, Department of Internal Medicine, Tri-Service General Hospital, National Defense Medical Center, Taipei, Taiwan, ROC; 5 Division of Nephrology, Department of Internal Medicine, Taoyuan Armed Forces General Hospital, Taoyuan City, Taiwan, ROC; Postgraduate Medical Institute, INDIA

## Abstract

Tinnitus mostly results from central and peripheral auditory pathology. Chronic kidney disease (CKD) is a major risk factor for cerebrovascular disease. However, no studies have evaluated the association between tinnitus and CKD. The aim of this study is to investigate the risk of tinnitus in patients with CKD. This retrospective cohort study was conducted using Taiwan National Health Insurance Research Database from 2000 to 2010. We established a CKD group (n = 185,430) and a non-CKD comparison group (n = 556,290) to investigate the incidence of tinnitus. Cox proportional hazard regression analysis was used to evaluate the effects of CKD on tinnitus risk. The results showed CKD significantly increased the risk of tinnitus (adjusted hazard ratio, 3.02; 95% CI, 2.655–3.456, P<0.001). A subgroup analysis revealed the increase in risk of tinnitus is more in CKD patients with heart failure (adjusted hazard ratio, 9.975; 95% CI, 5.001–18.752) and diabetes mellitus (adjusted hazard ratio, 3.712; 95% CI, 2.856–5.007). Furthermore, compared to non-CKD patients, the risk of tinnitus was increased 4.586-fold (95% CI, 2.399–6.7) in CKD patients with dialysis and 2.461-fold (95% CI, 1.033–3.454) in CKD patients without dialysis. This study is the first to report that CKD is associated with an increased risk of tinnitus. Among CKD cohort, patients with dialysis are at a higher risk of tinnitus than those without dialysis.

## Introduction

Chronic kidney disease (CKD) is characterized by progressive and irreversible loss of kidney function over the period of months and years. Diabetes and hypertension are the main causes in most countries and diabetes accounts for 30–50% of the patients with CKD [1]. Health-related quality of life in CKD population is poorer than general population and deteriorates as glomerular filtration rate declines. As CKD progresses, the formation and accumulation of uremic toxin will lead to adverse biological effects. Uremic toxin can contribute to inflammation, immune dysfunction, vascular disease and platelet dysfunction [1]. CKD is a major risk factor for many morbidities, including cardiovascular disease, cerebrovascular disease and bleeding, and for all-cause and cardiovascular mortality [1, 2]. An increased risk of death from cardiovascular disease rises with progressively worsening kidney function [2–4]. A strong inverse relationship between renal function and stroke is demonstrated and it shows that the risk of having stroke increases by 7% every 10 mL/min/1.73 m^2^ decline in estimated glomerular filtration rate (eGFR) [5]. In the patient with dialysis, stroke has a prevalence of 17% compared to 10% in non-dialysis patients and 4% in the general population [6, 7]. In addition, CKD increases risk of cognitive impairment by 65% and is related to hearing impairment [1, 8–10].

Tinnitus is a perception of sound in the absence of external auditory stimulus and often has a negative effect upon the quality of life. This symptom affects 5.1–42.7% of the general population [11]. Of those affected, approximately 3% to 5% suffer from severe troublesome tinnitus causing disability and disruption in daily performance [12]. Objective tinnitus results from the patient’s perception of an abnormal somatosound or abnormal sound perception. Subjective tinnitus, representing more than 90% of patients with tinnitus, is usually secondary to a sensorineural hearing loss and associated with emotional stress, depression, sleep disorder and anxiety [13–16]. Among the modalities of treatment, counselling, cognitive behavioral therapy, tinnitus maskers and hearing aids are more commonly used to reduce the distress associated with tinnitus. Medication is indicated only for the treatment of tinnitus-associated symptoms such as depression, sleep disturbances, and anxiety [14]. In addition to hearing loss, other factors such as diabetes, sleep apnea, previous head injuries, history of arthritis and hypertension have been suggested as risk factors [17–19]. Nevertheless, the association between CKD and the subsequent tinnitus risk has never been explored. Therefore we conducted a population based case-control study utilizing data from a nationwide health insurance database, the Taiwan National Health Insurance Research Database (NHIRD) to examine the risk of developing tinnitus among patients with CKD.

## Materials and methods

### Data sources

In this study, we used data from the NHIRD to investigate the risk of having tinnitus in patient with CKD over a 10-year period, from the outpatient Longitudinal Health Insurance Database in Taiwan (2000–2010), which is a valid representative sample of the total population of 23,000,000 in Taiwan. The National Health Insurance (NHI) Program was launched in Taiwan in 1995, and as of June 2009 it included contracts with 97% of the medical providers in Taiwan with approximately 23 million beneficiaries, or more than 99% of the entire population in Taiwan. [20] The NHIRD uses International Classification of Diseases, 9th Revision, Clinical Modification (ICD-9-CM) codes to record diagnoses. The Bureau of NHI randomly reviews the records of 1 in 100 ambulatory care visits and 1 in 20 in-patient claims to verify the accuracy of the diagnoses. Several studies have demonstrated the accuracy and validity of the diagnoses in the NHIRD [21–23]. The Institutional Review Board of Tri-Service General Hospital approved this study and waived the need for individual written informed consent (TSGH IRB No. 2-105-05-082).

### Study design and sampled participants

**Fig 1:**
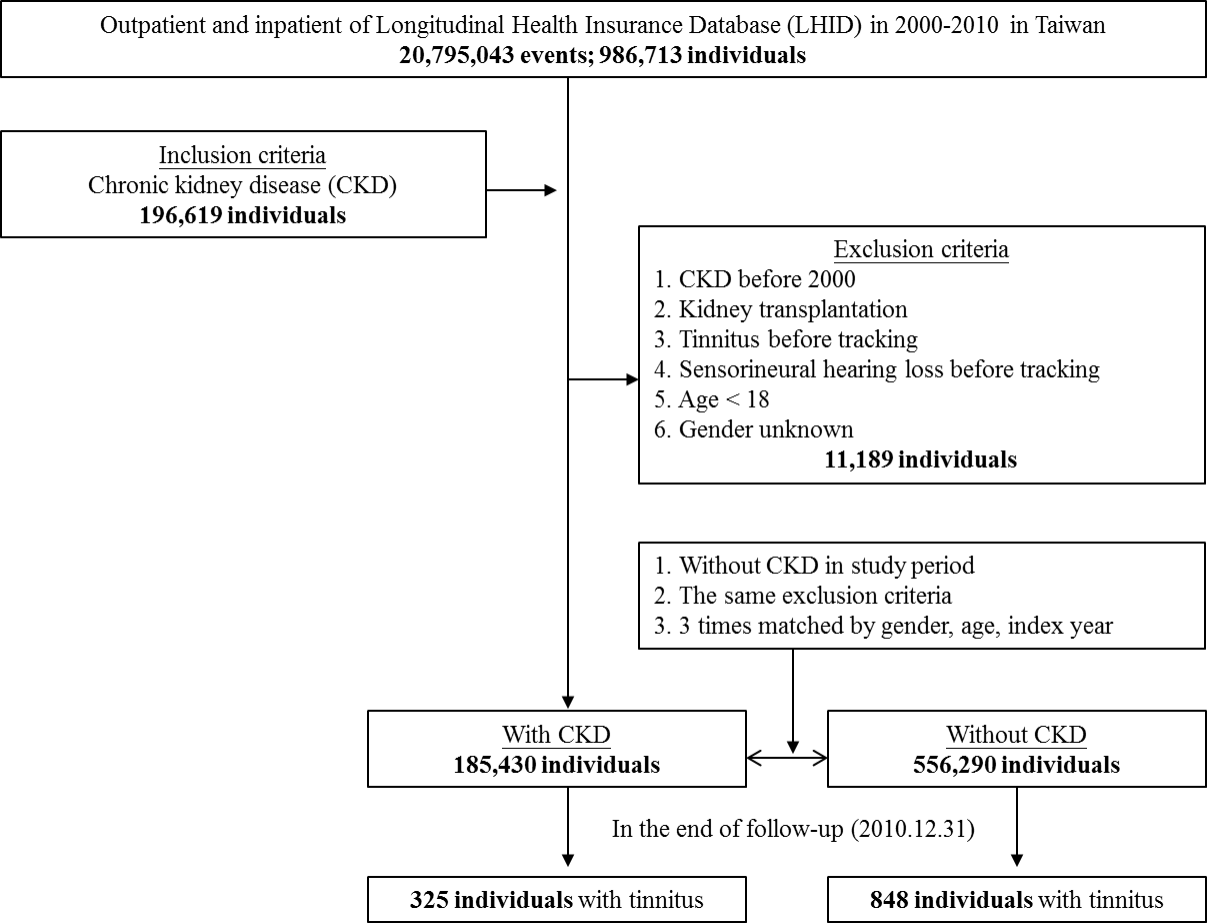
The flowchart of study sample selection from National Health Insurance Research Database in Taiwan.

This study was a retrospective matched-cohort design. Among the 986,713 individuals recorded in the outpatient and inpatient data from January 1, 2000 to December 31, 2010, 196,619 individuals were diagnosed as CKD (ICD-9-CM codes 585–586) prior to the index date. The patients were excluded if they were fit into one of the following criteria: newly diagnosed as CKD during the period of 1997–1999, history of receiving kidney transplantation (ICD-9-CM code V42.0), having tinnitus (ICD-9-CM code 388.3) or sensorineural hearing loss (ICD-9-CM code 389.1) before tracking, aged <18 years and gender unknown. Finally, the case group was 185,430 individuals. The control group had the same exclusion criteria of case group and without CKD in study period, and then we took 3 times matched by index year, index month, gender and age; that is, the control group was 556,290 individuals. The tracking of case and control groups continued until December 31, 2010. The event of tracking definite is the occurrence of tinnitus. The individuals having at least three times of diagnoses of tinnitus (ICD-9-CM code 388.3) by otolaryngologists, interspersed by a minimum of 4 weeks, were defined as developing tinnitus (Fig 1). Medical histories of drug intake in the individuals of this study were assessed. Intake of aminoglycosides, macrolides, loop diuretics, antineoplastic agents (consisting of platinum compounds, vinca alkaloids, bleomycin and chlorambucil), aspirin and NSAIDs was included in this analysis. A subgroup analysis was conducted to investigate the respective risk of tinnitus in CKD patients with dialysis and without dialysis.

The covariates included gender, age group (18–29, 30–39, 40–49, 50–59, ≥60 years), geographical area of residence (north, center, south, and east of Taiwan, and outlets islands), urbanization level of residence (level 1 to 4) and monthly income (in New Taiwan Dollars [NTD]; <18,000, 18,000–34,999, ≥35,000). The urbanization level of residence was defined according to the population and various indicators of the level of development. Level 1 was defined as a population >1,250,000, and a specific designation as political, economic, cultural and metropolitan development. Level 2 was defined as a population between 500,000 and 1249,999, and as playing an important role in the political system, economy, and culture. Urbanization levels 3 and 4 were defined as a population between 149,999 and 499,999, and <149,999, respectively.

Baseline comorbidities included hypertension (ICD-9-CM codes 401–405), diabetes mellitus (ICD-9-CM code 250), heart failure (ICD-9-CM code 428), stroke (ICD-9-CM codes 430–438), chronic obstructive pulmonary disease (ICD-9-CM codes 490–496), liver cirrhosis (ICD-9-CM code 571), Ménière’s disease (ICD-9-CM code 386.0), and traumatic brain injury (ICD-9-CM codes 800–804, 850–854, 873, 310.2). The Charlson comorbidity index after removal of the aforementioned comorbidities (CCI_R) was used to demonstrate the extent of the comorbidities.

### Statistical analysis

All analyses were performed using SPSS software version 22 (SPSS Inc., Chicago, Illinois, USA). χ^2^ and t tests were used to evaluate the distributions of categorical and continuous variables, respectively. Multivariate Cox proportional hazards regression analysis was used to determine the risk of tinnitus, and the results were present as hazard ratio (HR) with 95% confidence interval (CI). The difference in the risk of tinnitus between the study and control groups was estimated using the Kaplan-Meier method with the log-rank test. A 2-tailed P value less than 0.05 was considered to indicate statistical significance.

## Results

**Fig 2:**
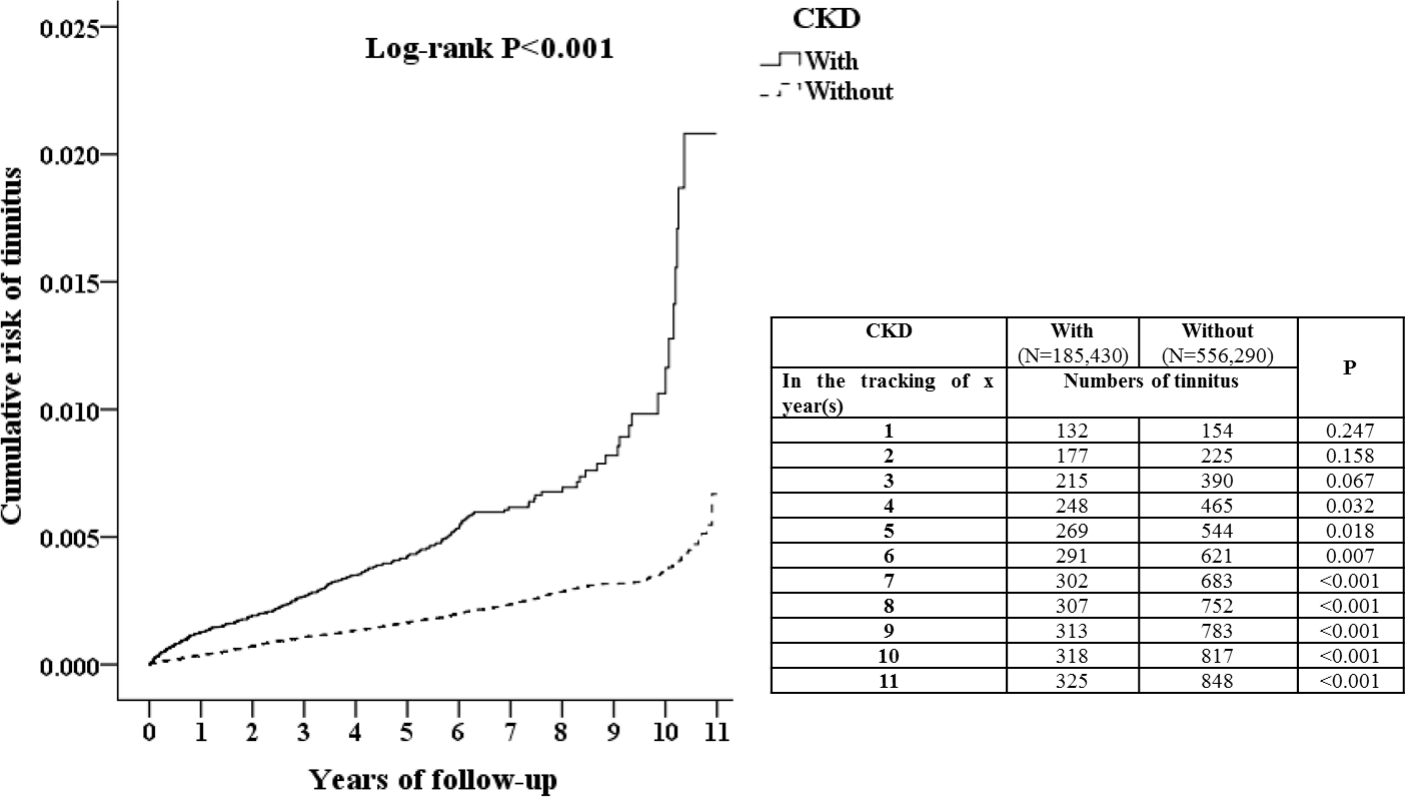
Kaplan-Meier for cumulative risk of tinnitus among patients aged 18 and over stratified by chronic kidney disease (CKD) with log-rank test.

This study examined 185,430 patients with CKD and 556,290 controls. Table 1 showed demographic characteristics of case and control groups. Compared to the controls, patients with CKD had higher rates of hypertension, diabetes mellitus, heart failure, liver cirrhosis and traumatic brain injury (P<0.001), and lower rates of stroke, chronic obstructive pulmonary disease and Ménière’s disease (P<0.001). The level of CCI_R was higher in CKD group than control group (P<0.001). Patients with CKD lived more in less urbanized areas and middle area of Taiwan (P<0.001). Over the 10-year follow-up, the cumulative incidence of developing tinnitus was 0.18% (325/185,430 individuals) in patients with CKD and 0.15% (848/556,290 individuals) in patients without CKD. The overall incidence of tinnitus was 97.25 per 100,000 person-years for patients with CKD and 72.56 per 100,000 person-years for patients without CKD. The Kaplan-Meier analysis indicated that patients with CKD had a significantly higher risk of developing tinnitus than did patients without CKD (log-rank test P<0.001) (Fig 2). At the 4^th^ year of follow-up, the difference between the two groups became significant (P = 0.032 in the tracking of 4 years). In addition, the incidence rate of sensorineural hearing loss at the end of follow-up was 65.2% in patients with CKD with tinnitus and 7.1% in individuals without CKD with tinnitus (P<0.001). CKD population had a higher association between tinnitus and sensorineural hearing loss. The Cox regression analysis of the factors associated with the risk of tinnitus showed the crude HR was 2.743 (95% CI = 2.404–3.13, P<0.001) (Table 2). After adjusting for age, gender, comorbidities, drug intake, geographical area of residence, urbanization level of residence and monthly income, the adjusted HR was 3.02 (95% CI = 2.655–3.456, P < .001). It indicated that patients with CKD had a 3.02 times higher risk of developing tinnitus compared to the individuals without CKD.

**Table 1:** Characteristics of study cohort in the baseline.

CKD	Total		With		Without		P
Variables	n	%	n	%	n	%	
**Total**	741,720		185,430	25.00	556,290	75.00	
**Gender**							0.999
Male	402,880	54.32	100,720	54.32	302,160	54.32	
Female	338,840	45.68	84,710	45.68	254,130	45.68	
**Age groups (years)**							0.999
18–29	10,226	1.38	2,559	1.38	7,667	1.38	
30–39	24,428	3.29	6,107	3.29	18,321	3.29	
40–49	63,152	8.51	15,788	8.51	47,364	8.51	
50–59	111,160	14.99	27,790	14.99	83,370	14.99	
≧60	532,744	71.83	133,186	71.83	399,558	71.83	
**Hypertension**							<0.001
Without	585,357	78.92	143,196	77.22	442,161	79.48	
With	156,363	21.08	42,234	22.78	114,129	20.52	
**Diabetes mellitus**							<0.001
Without	594,100	80.10	120,390	64.92	473,710	85.16	
With	147,620	19.90	65,040	35.08	82,580	14.84	
**Heart failure**							<0.001
Without	702,944	94.77	163,989	88.44	538,955	96.88	
With	38,776	5.23	21,441	11.56	17,335	3.12	
**Stroke**							<0.001
Without	672,258	90.64	172,250	92.89	500,008	89.88	
With	69,462	9.36	13,180	7.11	56,282	10.12	
**COPD**							<0.001
Without	675,601	91.09	173,503	93.57	502,098	90.26	
With	66,119	8.91	11,927	6.43	54,192	9.74	
**Liver cirrhosis**							<0.001
Without	699,834	94.35	174,520	94.12	525,314	94.43	
With	41,886	5.65	10,910	5.88	30,976	5.57	
**Ménière’s disease**							<0.001
Without	727,830	98.13	183,996	99.23	543,834	97.76	
With	13,890	1.87	1,434	0.77	12,456	2.24	
**Traumatic brain injury**							<0.001
Without	637,875	86.00	133,055	71.75	504,820	90.75	
With	103,845	14.00	52,375	28.25	51,470	9.25	
**CCI_R**	1.15±2.07		2.61±1.65		0.67±1.97		<0.001
**Drug intake**							
**Aminoglycosides**							<0.001
Without	731,967	98.69	183,189	98.79	548,778	98.65	
With	9,753	1.31	2,241	1.21	7,512	1.35	
**Macrolides**							<0.001
Without	734,112	98.97	183,374	98.89	550,738	99.00	
With	7,608	1.03	2,056	1.11	5,552	1.00	
**Loop diuretics**							0.462
Without	734,448	99.02	183,585	99.01	550,863	99.02	
With	7,272	0.98	1,845	0.99	5,427	0.98	
**Antineoplastic agents**							<0.001
Without	734,719	99.06	183,444	98.93	551,275	99.10	
With	7,001	0.94	1,986	1.07	5,015	0.90	
**Aspirin**							0.076
Without	737,636	99.45	184,458	99.48	553,178	99.44	
With	4,084	0.55	972	0.52	3,112	0.56	
**NSAIDs**							<0.001
Without	733,020	98.83	183,418	98.91	549,602	98.80	
With	8,700	1.17	2,012	1.09	6,688	1.20	
**Location**							<0.001
Northern Taiwan	285,200	38.45	66,494	35.86	218,706	39.32	
Middle Taiwan	216,509	29.19	59,579	32.13	156,930	28.21	
Southern Taiwan	190,913	25.74	47,140	25.42	143,773	25.84	
Eastern Taiwan	45,317	6.11	11,170	6.02	34,147	6.14	
Outlets Islands	3,781	0.51	1,047	0.56	2,734	0.49	
**Urbanization level**							<0.001
1 (The highest)	235,150	31.70	54,427	29.35	180,723	32.49	
2	327,839	44.20	83,352	44.95	244,487	43.95	
3	54,083	7.29	12,920	6.97	41,163	7.40	
4 (The lowest)	124,648	16.81	34,731	18.73	89,917	16.16	
**Insured premium (NT$)**							<0.001
<18,000	732,832	98.80	183,435	98.92	549,397	98.75	
18,000–34,999	7,780	1.05	1,788	0.96	5,992	1.08	
≧35,000	1,158	0.16	207	0.11	951	0.17	

P-value (category variable: Chi-square/Fisher exact test; continue variable: t-test); COPD = chronic obstructive pulmonary disease; CCI_R = Charlson comorbidity index with removal of the aforementioned comorbidities

**Table 2:** Factors of tinnitus in the end of follow-up by using Cox regression.

Variables	Crude HR	95% CI	95% CI	P	Adjusted HR	95% CI	95% CI	P
**CKD**								
Without	Reference				Reference			
With	2.743	2.404	3.130	<0.001	3.020	2.655	3.456	<0.001
**Gender**								
Male	0.951	0.848	1.067	0.393	0.981	0.854	1.115	0.689
Female	Reference				Reference			
**Age groups (years)**								
18–29	Reference				Reference			
30–39	0.754	0.334	1.703	0.497	0.753	0.355	1.642	0.454
40–49	0.935	0.450	1.945	0.585	0.901	0.444	1.978	0.789
50–59	0.884	0.435	1.798	0.734	0.896	0.498	1.889	0.750
≧60	0.562	0.280	1.128	0.105	0.675	0.311	1.598	0.324
**Hypertension**								
Without	Reference				Reference			
With	1.079	0.955	1.220	0.222	1.012	0.815	1.154	0.313
**Diabetes mellitus**								
Without	Reference				Reference			
With	0.905	0.789	1.039	0.155	0.856	0.711	0.998	0.048
**Heart failure**								
Without	Reference				Reference			
With	0.582	0.425	0.745	<0.001	0.557	0.411	0.754	<0.001
**Stroke**								
Without	Reference				Reference			
With	0.892	0.739	1.077	0.234	0.981	0.655	1.227	0.557
**COPD**								
Without	Reference				Reference			
With	0.905	0.752	1.090	0.293	1.101	0.876	1.468	0.264
**Liver cirrhosis**								
Without	Reference				Reference			
With	1.071	0.834	1.376	0.591	1.111	0.875	1.498	0.483
**Ménière’s disease**								
Without	Reference				Reference			
With	8.137	6.723	9.850	<0.001	8.100	6.666	9.879	<0.001
**Traumatic brain injury**								
Without	Reference				Reference			
With	0.822	0.672	1.005	0.056	0.797	0.654	0.990	0.045
**CCI_R**	0.956	0.934	0.982	0.001	0.978	0.901	0.988	0.003
**Drug intake**								
**Aminoglycosides**								
Without	Reference				Reference			
With	1.154	0.495	2.267	0.226	1.135	0.546	2.198	0.271
**Macrolides**								
Without	Reference				Reference			
With	1.598	0.387	2.975	0.304	1.448	0.402	2.745	0.331
**Loop diuretics**								
Without	Reference				Reference			
With	0.989	0.445	1.577	0.745	0.977	0.304	1.368	0.798
**Antineoplastic agents**								
Without	Reference				Reference			
With	0.950	0.108	1.998	0.672	0.931	0.101	1.875	0.842
**Aspirin**								
Without	Reference				Reference			
With	1.372	0.562	2.245	0.775	1.401	0.333	2.298	0.811
**NSAIDs**								
Without	Reference				Reference			
With	0.997	0.334	1.765	0.842	1.013	0.297	1.865	0.765
**Location**					Had collinearity with urbanization level			
Northern Taiwan	Reference				Had collinearity with urbanization level			
Middle Taiwan	1.272	1.102	1.468	0.001	Had collinearity with urbanization level			
Southern Taiwan	1.167	1.003	1.358	0.045	Had collinearity with urbanization level			
Eastern Taiwan	1.696	1.377	2.089	<0.001	Had collinearity with urbanization level			
Outlets Islands	1.375	0.614	3.079	0.439	Had collinearity with urbanization level			
**Urbanization level**								
1 (The highest)	1.249	1.050	1.486	0.012	1.145	0.943	1.389	0.171
2	1.070	0.907	1.264	0.421	0.986	0.827	1.174	0.872
3	1.251	0.976	1.602	0.077	1.236	0.965	1.584	0.093
4 (The lowest)	Reference				Reference			
**Insured premium (NT$)**								
<18,000	Reference				Reference			
18,000–34,999	1.521	1.007	2.299	0.048	1.389	0.919	2.101	0.119
≧35,000	0.667	0.094	4.738	0.688	0.514	0.072	3.658	0.506

HR = hazard ratio; CI = confidence interval; Adjusted HR = adjusted variables listed in the table; COPD = chronic obstructive pulmonary disease; CCI_R = Charlson comorbidity index with removal of the aforementioned comorbidities

In the subgroups stratified by the gender, age, comorbidities, drug intake, urbanization and monthly income, the CKD individuals who were female, aged <30 years and in the lowest urbanization level were associated with a higher risk of developing tinnitus than those who were male, aged ≥30 years and in higher urbanization level (Table 3). Those with hypertension, diabetes mellitus, heart failure, chronic obstructive pulmonary disease, liver cirrhosis and traumatic brain injury were associated with a higher risk of developing tinnitus than those without these comorbidities. The adjusted HR of tinnitus was 3.712 (95% CI, 2.856–5.007) in those with diabetes mellitus and 2.788 (95% CI, 2.012–3.864) in those without diabetes mellitus. The adjusted HR of tinnitus was 9.975 (95% CI, 5.001–18.752) in those with heart failure and 2.82 (95% CI, 2.012–3.336) in those without heart failure. A significant association between CKD and tinnitus development was observed among CKD patients with drug intake compared with the controls with drug intake (Table 3). CKD patients with intake of aminoglycosides, macrolides, loop diuretics, antineoplastic agents, aspirin and NSAIDs exhibited a higher risk of developing tinnitus than controls with intake of those drugs (adjusted HR: 2.895 in those using aminoglycosides, 2.765 in those using macrolides, 2.986 in those using loop diuretics, 2.901 in those using antineoplastic agents, 2.872 in those using aspirin, and 2.601 in those using NSAIDs). It revealed that CKD was a significantly risk factor for tinnitus in the individuals with intake of ototoxic agents. Among CKD population (185,430 individuals), there were patients with dialysis (87,361 individuals, 47.11%) and without dialysis (98,069 individuals, 52.89%). The incidence and HR of tinnitus in CKD population with or without dialysis relative to those in controls were listed in Table 4. The risk of tinnitus was 4.586-fold (95% CI, 2.399–6.7) higher in patients with CKD and dialysis, and was 2.461-fold (95% CI, 1.033–3.454) higher in patients with CKD and without dialysis. Among CKD population, cases with dialysis carried a higher risk of developing tinnitus than those without dialysis.

**Table 3:** Factors of tinnitus in the end of follow-up stratified by variables listed in the table by using Cox regression.

CKD	With			Without			Ratio	Adjusted HR	95%CI	95%CI	P
Variables	Event	PYs	Rate	Event	PYs	**Rate**					
**Total**	325	334,193.43	97.25	848	1,168,647.66	72.56	1.340	3.020	2.655	3.456	<0.001
**Gender**											
Male	166	174,093.39	95.35	449	629,014.38	71.38	1.336	2.992	2.455	3.712	<0.001
Female	159	160,100.04	99.31	399	539,633.28	73.94	1.343	3.111	2.564	3.672	<0.001
**Age groups (years)**											
18–29	6	2,450.49	244.85	2	4,350.17	45.98	5.326	4.311	0.636	29.875	0.234
30–39	8	8,722.15	91.72	13	15,192.58	85.57	1.072	2.189	0.745	5.871	0.126
40–49	24	24,547.77	97.77	45	69,293.14	64.94	1.505	1.755	0.985	3.121	0.059
50–59	42	54,197.86	77.49	125	305,306.51	40.94	1.893	1.311	0.911	1.886	0.265
≧60	245	244,275.16	100.30	663	744,505.26	89.05	1.126	3.789	3.295	4.457	<0.001
**Hypertension**											
Without	193	196,821.62	98.06	598	823,506.35	72.62	1.350	2.999	2.565	3.572	<0.001
With	132	137,371.81	96.09	250	345,141.31	72.43	1.327	3.174	2.511	4.065	<0.001
**Diabetes mellitus**											
Without	213	226,747.49	93.94	699	902,614.94	77.44	1.213	2.788	2.012	3.864	<0.001
With	112	107,445.94	104.24	149	266,032.72	56.01	1.861	3.712	2.856	5.007	<0.001
**Heart failure**											
Without	293	302,066.10	97.00	829	1,085,462.92	76.37	1.270	2.820	2.012	3.336	<0.001
With	32	32,127.33	99.60	19	83,184.74	22.84	4.361	9.975	5.001	18.752	<0.001
**Stroke**											
Without	299	303,790.75	98.42	753	1,031,091.16	73.03	1.348	3.099	2.612	3.981	<0.001
With	26	30,402.68	85.52	95	137,556.50	69.06	1.238	2.701	1.310	4.412	<0.001
**COPD**											
Without	306	313,272.56	97.68	742	1,024,602.66	72.42	1.349	3.001	2.654	3.596	<0.001
With	19	20,920.87	90.82	106	144,045.00	73.59	1.234	3.085	1.777	4.896	<0.001
**Liver cirrhosis**											
Without	304	317,482.25	95.75	804	1,108,300.52	72.54	1.320	2.984	2.211	3.598	<0.001
With	21	16,711.18	125.66	44	60,347.14	72.91	1.724	3.611	1.975	5.565	<0.001
**Ménière’s disease**											
Without	310	331,468.23	93.52	746	1,131,989.89	65.90	1.419	3.058	2.672	3.856	<0.001
With	15	2,725.20	550.42	102	36,657.77	278.25	1.978	2.892	1.311	4.750	0.002
**Traumatic brain injury**											
Without	268	274,340.62	97.69	801	1,057,755.74	75.73	1.290	2.989	2.311	3.745	<0.001
With	57	59,852.81	95.23	47	110,891.92	42.38	2.247	3.912	2.711	6.201	<0.001
**Drug intake**											
**Aminoglycosides**											
Without	266	274,647.98	96.85	759	1,072,193.54	70.79	1.368	3.212	1.986	4.895	0.002
With	59	59,545.45	99.08	89	96,454.12	92.27	1.074	2.895	1.012	3.898	0.025
**Macrolides**											
Without	280	275,740.83	101.54	779	1,078,193.64	72.25	1.405	3.336	2.015	4.871	<0.001
With	45	58,452.60	76.99	69	90,454.02	76.28	1.009	2.765	1.008	5.986	0.044
**Loop diuretics**											
Without	227	252,680.41	89.84	684	1,005,196.49	68.05	1.320	3.121	2.298	5.156	0.001
With	98	81,513.02	120.23	164	163,451.17	100.34	1.198	2.986	1.145	4.996	0.039
**Antineoplastic agents**											
Without	248	262,936.56	94.32	716	1,030,243.76	69.50	1.357	3.201	2.454	5.986	0.011
With	77	71,256.87	108.06	132	138,403.90	95.37	1.133	2.901	1.065	7.454	0.040
**Aspirin**											
Without	257	263,498.05	97.53	737	1,046,751.39	70.41	1.385	3.345	1.806	8.975	<0.001
With	68	70,695.38	96.19	111	121,896.27	91.06	1.056	2.872	1.012	6.154	0.035
**NSAIDs**											
Without	241	249,346.68	96.65	695	1,004,186.68	69.21	1.397	3.465	1.445	7.565	0.012
With	84	84,846.75	99.00	153	164,460.98	93.03	1.064	2.601	1.111	5.972	0.038
**Urbanization level**											
1 (The highest)	106	100,886.42	105.07	271	335,111.42	80.87	1.299	2.912	2.131	3.611	<0.001
2	147	153,565.48	95.72	364	533,090.84	68.28	1.402	3.098	2.442	3.950	<0.001
3	24	22,041.58	108.89	69	84,000.90	82.14	1.326	2.804	1.601	4.765	<0.001
4 (The lowest)	48	57,699.95	83.19	144	216,444.50	66.53	1.250	3.111	2.295	4.895	<0.001
**Insured premium (NT$)**											
<18,000	317	329,339.72	96.25	832	1,151,799.52	72.23	1.333	3.001	2.644	3.795	<0.001
18,000–34,999	8	4,398.00	181.90	15	15,336.07	97.81	1.860	5.542	2.011	16.712	<0.001
≧35,000	0	455.71	0	1	1,512.07	66.13	0	0	-	-	0.989

PYs = person-years; Rate = incidence rate (per 100,000 person-years); Ratio = rate in with ÷ rate in without; Adjusted HR = adjusted hazard ratio: adjusted variables listed in Table 2; CI = confidence interval; CCI_R = Charlson comorbidity index with removal of the aforementioned comorbidities.

**Table 4:** Comparison of incidence and HR of tinnitus between patients with CKD with and without dialysis and patients without CKD.

Variable	n	Event	PYs	Rate	Adjusted HR (95% CI)
**Without CKD**	556,290	848	1,168,647.66	72.6	Reference
**With CKD**					
**Dialysis**	87,361	192	133,761.97	143.5	4.586 (2.399–6.700)*
**Non-dialysis**	98,069	133	200,431.46	66.4	2.461 (1.033–3.454)**

HR = hazard ratio; CKD = chronic kidney disease; PYs = person-years; Rate = incidence rate (per 100,000 person-years); CI = confidence interval; Adjusted HR = adjusted hazard ratio: adjusted variables listed in Table 2. Significance

*P < .05

**P < .001

## Discussion

This nationwide, population-based study is the first research to investigate the association between tinnitus and CKD. We confirm CKD is a significant risk factor for tinnitus. The CKD population is at a 3.02 times higher risk of tinnitus compared to the general population. Among CKD population, the patients who are female and aged <30 years have a higher risk of developing tinnitus than those who are male and aged ≥30 years. In addition, the patients with hypertension, diabetes mellitus, heart failure, liver cirrhosis and traumatic brain injury carry 3.174–9.975 times risk of tinnitus. Therefore hypertension, diabetes mellitus, heart failure, liver cirrhosis and traumatic brain injury are contributing factors to develop tinnitus in patients with CKD. In this study, another important finding is that the patients with hemodialysis or peritoneal dialysis have a higher risk of developing tinnitus than those without dialysis. It indicates that the patients with end stage renal disease carry a more increased risk of developing tinnitus. It also suggests the risk of developing tinnitus is positively correlated to the severity of the loss of kidney function.

The pathophysiological mechanism of tinnitus is complex and multifactorial. The occurrence of tinnitus is often related to hearing malfunction. The well recognized mechanism is downregulation of intracortical inhibition related to the damage to cochlea [24]. Diminished output from the damaged cochlea causes diminished inhibition in central auditory structures. The elevated multiunit spontaneous activity detected in dorsal cochlear nucleus can project to higher auditory centers. It eventually leads to activation of the auditory perceptual machinery, including primary auditory cortex, responsible for tinnitus [25]. The subtotal cochlear damage without changes of hearing threshold, a possible cause of tinnitus in patients without detectable hearing loss, is linked to a degeneration of auditory neuron with damage of inner hair cell synapse. This kind of auditory deprivation can result to a failure to appropriately adapt the central response gain, which may cause tinnitus [26]. Neural synchrony and tonotopic map reorganization in the auditory modality are two other possible mechanisms in the occurrence of tinnitus after hearing loss [17]. The negative impact of chronic tinnitus has been recognized due to neuronal connectivity and interactions between auditory and limbic systems [27]. The functional neuroimaging studies revealed subjects with tinnitus had abnormal functioning both within the central auditory system and in non-auditory brain areas. The neural activity in non-auditory areas including the frontal areas, the limbic system and the cerebellum seems be associated with the perception of tinnitus [28]. The change in limbic system is contributed to the perception and annoyance of tinnitus, and possibly to the development of tinnitus.

Although the physiological mechanisms behind the association between CKD and the subsequent occurrence of tinnitus remain unclear, we inferred potential mechanisms from previous studies that may provide possible explanations. First, two population-based studies demonstrate the association between hearing loss and CKD [8, 10]. Vilayur et al. report that moderate CKD is independently associated with hearing loss [8]. Subjects with eGFR < 45 mL/min/1.73 m^2^ have the highest prevalence of hearing loss. Seo at al. report that the odds ratio of hearing impairment was 1.25 times higher in subjects with eGFR < 60 mL/min/1.73 m^2^ than in those with eGFR ≥ 60 mL/min/1.73 m^2^ [10]. A combination of factors resulting from CKD, including abnormal electrolytes, urea, and creatinine levels, can lead to the cochlear dysfunction [10]. The cochlea and kidney have similar physiological mechanisms, including the active transport of fluid and electrolytes in stria vascularis of cochlea and glomerulus of kidney [29]. Thus the nephrotoxic substances and medication, an important cause of CKD, may result in the cochlear damage. CKD is associated with an increased inflammatory status [30]. Inflammation is proposed as an etiopathogenic mechanism of hearing loss in chronic nephropathy because of the induction of cochlear microcirculation dysfunction [31]. In addition, CKD is a risk factor for the occurrence of cerebral small vessel disease. It may disturb the blood supply to the inner ear and lead to the cochlear ischemic damage [32]. Overall, the CKD-related cochlear dysfunction or auditory deafferentation can be attributed to the development of tinnitus. Second, the patients with tinnitus usually presents with the activation of limbic system [33, 34]. Palkovits et al. find the activation of neuron within the limbic and stress-related areas in a rat model of mild chronic renal failure [35]. The activation of limbic system related to CKD is another possible mechanism to induce tinnitus. Third, hyperactivity of the sympathetic nerve system in patients with renal failure was reported [36, 37]. An increase in sympathetic activity can play a role in the pathogenesis of tinnitus.

The strengths of this study are its population-based research, use of well-established cohort data with a large sample size and extended follow-up period to identify CKD as a risk factor of developing tinnitus. Nevertheless, there are several limitations to this study. First, although the coding of the NHIRD in recording a few diseases has been validated, no reports are available regarding the coding accuracy for tinnitus. Nevertheless, we have restricted the diagnoses of tinnitus to only those made by otolaryngologists to increase its validity. Second, patients with tinnitus can be identified using the insurance claims data, however the information on tinnitus handicap is not available. Therefore, the effect of CKD on the severity of tinnitus cannot be analyzed. Third, the incidence of tinnitus may be underestimated because patients with mild and acceptable tinnitus cannot be clearly identified from the Taiwan NHI data set.

## Conclusions

This study presented that CKD is a significant and independent risk factor for tinnitus. The patients with CKD have a 3.02 times higher risk of developing tinnitus. Furthermore the patients with end stage renal disease and dialysis are at a 4.586 times risk of tinnitus than general population and carry a higher risk of tinnitus than the patients with CKD and without dialysis. The underlying mechanism of this association and the effect of disease progression on the development of tinnitus need clarifying in future studies.
